# Factors associated with post-diagnosis pregnancies in women living with HIV in the south of Brazil

**DOI:** 10.1371/journal.pone.0172514

**Published:** 2017-02-21

**Authors:** Luciana Barcellos Teixeira, Flávia Bulegon Pilecco, Álvaro Vigo, Maria de Lourdes Drachler, José Carlos de Carvalho Leite, Daniela Riva Knauth

**Affiliations:** 1 Department of Professional Assistance and Guidance, Nursing School, Federal University of Rio Grande do Sul (Universidade Federal do Rio Grande do Sul), Porto Alegre, Rio Grande do Sul, Brazil; 2 Graduate Studies Program in Public Health, Federal University of Rio Grande do Sul (Universidade Federal do Rio Grande do Sul), Porto Alegre, Rio Grande do Sul, Brazil; 3 Department of Social Medicine, Federal University of Rio Grande do Sul (Universidade Federal do Rio Grande do Sul), Porto Alegre, Rio Grande do Sul, Brazil; 4 Graduate Studies Program in Epidemiology, Federal University of Rio Grande do Sul (Universidade Federal do Rio Grande do Sul), Porto Alegre, Rio Grande do Sul, Brazil; 5 Department of Statistics, Federal University of Rio Grande do Sul (Universidade Federal do Rio Grande do Sul), Porto Alegre, Rio Grande do Sul, Brazil; 6 University Center UNILASALLE, Rio Grande do Sul, Brazil; Indiana University, UNITED STATES

## Abstract

**Objectives:**

To analyze the factors associated with the occurrence of pregnancies after the diagnosis of infection by HIV.

**Methods:**

Cross-sectional study with women of a reproductive age living with HIV/AIDS cared for in the public services of the city of Porto Alegre, in southern Brazil. The data was analyzed from a comparison between two groups: women with and women without pregnancies after the diagnosis of HIV. Poisson regression models were used to estimate the reasons of prevalence (RP).

**Results:**

The occurrence of pregnancies after the diagnosis of HIV is associated with a lower level of education (RP adjusted = 1.31; IC95%: 1.03–1.66), non-use of condoms in the first sexual intercourse (RP = 1.32; IC95%: 1.02–1.70), being 20 years old or less when diagnosed with HIV (RP = 3.48; IC95%: 2.02–6.01), and experience of violence related to the diagnosis of HIV (RP = 1.28; IC95%: 1.06–1.56).

**Conclusions:**

The occurrence of pregnancies after the diagnosis of infection by HIV does not indicate the exercise of the reproductive rights of the women living with HIV/AIDS because these pregnancies occurred in contexts of great vulnerability.

## Introduction

In the world, women total approximately 50% of the cases of infection by HIV. In Latin America, they represent 36% of the adults living with HIV/AIDS [1]. In Brazil, women account for 35% of the cases of AIDS registered in the period from 1980 to July 2014, of which 50% were between 25 to 39 years old [2]. The reduction of the risk of the vertical transmission of HIV by the adoption of the ACTG 076 [3–5] protocol and the treatment of children exposed to HIV, as well as the fundamentals for the control of the epidemic [6], can provide security and increase the possibilities of the exercise of reproductive rights by women living with HIV.

Several studies have investigated the impact of the diagnosis of infection by HIV in the reproductive decisions of women [7–11] and in the rates of fertility and of sterilization [12–13]. American women living with HIV/AIDS present lower rates of fertility and higher rates of sterilization than women without this diagnosis [12]. Also, in a Brazilian study, the rates of sterilization were higher among women living with HIV/AIDS compared to the female population in general [13].

As well as the impact of the diagnosis, the prophylaxis of the vertical transmission can also influence the reproductive decisions of women living with HIV/AIDS [14,15]. Women of reproductive age who discover that they are seropositive for HIV face the difficult decision to have children or not. This decision is even more difficult in societies in which the appreciation of women is linked to maternity [16]. Studies in different contexts demonstrate that the reproductive decisions of women are heavily influenced by the significance that is culturally given to maternity [17–19].

The sexual and reproductive health of women living with HIV/AIDS comprises both the general aspect of being a woman and the particularities of the experience related to the virus [20,21]. In fact, decisions related to having or not having children are a right of women living with HIV/AIDS, influenced by social and cultural aspects. This article has the objective of analyzing the factors associated with the occurrence of pregnancies after the diagnosis of infection by HIV in women residing in southern Brazil.

## Materials and methods

The analyzed data is derived from a cross-sectional study entitled “Sexual and reproductive health of women in the context the epidemic of HIV/AIDS in Porto Alegre”. Data collection took place from January to November 2011. The study population was women of a reproductive age (18 to 49 years old) living with HIV/AIDS and cared for by specialized services in Porto Alegre. The sample size was calculated seeking to estimate the reasons of prevalence of pregnancy after the diagnosis of HIV of 1.6 or greater. This sample was estimated for an expected prevalence of the outcome between 30 and 70% in the group without the interest factor. A power of 80% and a bilateral statistical significance of 5% were considered. The calculation was performed in the PROC POWER procedure of the SAS (Statistical Analysis System version 9.2) program. Applying the correction of the effect of the outlining by complex sampling (deff) equal to 1.6, the final sample size was estimated in 615 women with HIV.

All specialized outpatient services in HIV/AIDS in the city of Porto Alegre in 2011 were included in the study. A simple random sampling was carried out at each service for women from 18 to 49 years old, respecting the proportionality of care of the services. The selection was based on the consultation appointment schedules of all health professionals of the service.

After reading and signing the informed voluntary consent form, the participants responded to a computer questionnaire prepared by the Sphinks Léxica version 4.0 program, in a private location, filled out by the interviewer on a netbook. The questionnaire investigated the following variables: (a) occurrence of pregnancies after the diagnosis of HIV, which is the outcome of interest for this study; and (b) socio-demographical and behavioral variables, including race-ethnicity, level of education, household income (measured by the minimum wage unit), age at diagnosis of infection by HIV, time of diagnosis of infection by HIV, use of condoms and age of first sexual intercourse, number of sexual partners and stable unions in life, practice of sex for money, use of illegal drugs, occurrence of children before and after the diagnosis of HIV, experience of violence related to the diagnosis of HIV/AIDS (either verbal or physical aggression related to HIV or occurrence of discrimination in health care services). To control the quality of the data, 10% of the questionnaires were randomly selected and repeated by telephone. The study was approved by the following ethics committees: Comitê de Ética em Pesquisa da Universidade do Rio Grande (UFRGS—approval number 2008216), Comitê de Ética em Pesquisa da Secretaria Municipal de Saúde de Porto Alegre, Comitê de Ética do Grupo Hospital Conceição, Comitê de Ética em Pesquisa do Hospital de Clínicas de Porto Alegre and the Comitê de Ética em Pesquisa da Secretaria Estadual de Saúde do Rio Grande do Sul.

The management of the database and the statistical analyses were performed using SPSS Software version 18.0. The characteristics of the sample are presented by descriptive statistics. The test of homogeneity of proportions, based on the Pearson chi-squared statistic, was used to compare proportions of exploratory variables between defined groups by the outcome of interest (women with and without pregnancies after the diagnosis of HIV), with a defined level of significance of 5%.

Poisson regression models with a strong estimator were used to estimate reasons of prevalence (RP) for each category of the exploratory variables, having as a reference the category of least expected risk. The modelling technique estimated the reasons of raw prevalence for each exploratory variable, using the Wald statistic, with a level of significance of 5%. Subsequently, adjusted reasons of prevalence were calculated in a regression model that simultaneously included all the variables that presented a value p ≤ 0.20. This model was used to examine the independent effect of each exploratory variable regarding the outcome, accordingly identifying the main predictors of the outcome, those whose value p was ≤ 0.05 using the Wald statistic. The time of diagnosis variable was included in the modelling as a possible confounding factor because it could influence both the exposition variables and the outcome of the study.

## Results

756 women from 18 to 49 years old living with HIV and cared for in the specialized outpatient services were selected as participants of the study. Of these women, 75 women refused to participate in this study. Therefore, the final sample was composed of 681 women; 90.1% of the 756 women who met the criteria for inclusion.

Table 1 presents the socioeconomic characteristics and experiences of women living with HIV/AIDS, in accordance with the occurrence of pregnancies after the diagnosis of HIV/AIDS. From the total of interviewed women, 35.2% reported pregnancies after the diagnosis of infection by HIV. The total sample consisted predominantly of white women (59.2%), with a level of education below high school (66.5%), a household income of less than two minimum salaries (53.2%) and at least two stable unions in life (58.2%). The use of illegal drugs in their lives was reported by 30% of the sample and 37.5% of them reported experience of violence related to the diagnosis of HIV/AIDS. The diagnosis of HIV/AIDS occurred at the age of up to 25 in 39.5% of the women (the mean age at diagnosis was 28,49 ± 7,57 years).

**Table 1:** Socioeconomic characteristics and experiences of women living with HIV, in accordance with the occurrence of pregnancies after diagnosis, in Porto Alegre, 2011.

Characteristics	All	Women with pregnancies after the diagnosis of HIV∕Aids^a^	Women without pregnancies after the diagnosis of HIV∕Aids^a^	p ^b^
**Age at diagnosis of HIV/Aids** ^c^				<0.001
≤ 20	109 (16%)	65 (27.2%)	44 (10%)	
21–25	160 (23.5%)	79 (33.1%)	81 (18.4%)	
26–30	151 (22.2%)	57 (23.8%)	94 (21.3%)	
31–35	116 (17.1%)	23 (9.6%)	93 (21.1%)	
≥ 36	144 (21.2%)	15 (6.3%)	129 (29.3%)	
**Race ∕ethnicity (self-declared)**				0.190
White	403 (59.2%)	134 (55.8%)	269 (61%)	
Others	278 (40.8%)	106 (44.2%)	172 (39%)	
**Level of education**				0.002
< High school	453 (66.5%)	178 (74.2%)	275 (62.4%)	
≥ High school	228 (33.5%)	62 (25.8%)	166 (37.6%)	
**Household income (in minimum salaries)**^d^				0.040
< 2	345 (53.2%)	135 (58.7%)	210 (50.2%)	
≥ 2	303 (46.8%)	95 (41.3%)	208 (49.8%)	
**Number of stable unions**				0.069
0	42 (6.2%)	12 (5%)	30 (6.8%)	
1	243 (35.7%)	73 (30.4%)	170 (38.6%)	
2 or more	396 (58.2%)	155 (64.6%)	240 (54.5%)	
**Use of illegal drugs during their lives**				0.001
Yes	203 (30%)	91 (38.1%)	122 (25.6%)	
No	474 (70%)	148 (61.9%)	326 (74.4%)	
**Experience de violence related to the diagnosis of HIV/Aids**				<0.001
Yes	254 (37.5%)	122 (51.3%)	132 (30%)	
No	424 (62.5%)	116 (48.7%)	308 (70%)	
**Total**	**681 (100%)**	**240 (100%)**	**441 (100%)**	

^a^ Totals may differ due to the possibility of non-response.

^b^ Value p associated to the test of homogeneity of proportions based on the Pearson chi-squared statistic.

^c^ Mean ± standard deviation = 28,49±7,57 years, 24,54 ± 6,14 years in the group of Women with pregnancies after the diagnosis of HIV∕Aids and 30,63 ± 6,14 in the group of Women without pregnancies after the diagnosis of HIV∕Aids.

^d^ The minimum salary at the time was R$ 545.00 = $ 349.38 (converted on July 1, 2011).

Table 2 presents the sexual and reproductive behavior of women, demonstrating that 47.1% of the sample had their first sexual initiation at up to 15 years of age (the mean age at the first sexual intercourse was 16,10 ± 2,73 years) and that 74.5% of the interviewees did not use condoms in their first sexual intercourse. The median of the number of sexual partners throughout life was 5, with partners ranging from 1 to 1300, and 33.3% of women had 7 or more partners. The practice of sex in exchange for money was reported by 11.2% of women and 69.7% of the sample already had a child before the diagnosis.

**Table 2:** Sexual and reproductive behavior of women living with HIV, in accordance with the occurrence of pregnancies after diagnosis, in Porto Alegre, 2011.

Characteristics	All	Women with pregnancies after the diagnosis of HIV∕Aids^a^	Women without pregnancies after the diagnosis of HIV∕Aids^a^	p^b^
**Age at first sexual intercourse**^c^				0.001
≤15 years	320 (47.1%)	133 (55.6%)	187 (42.5%)	
˃15 years	359 (52.9%)	106 (44.4%)	253 (57.5%)	
**Use of condom in the first sexual intercourse**				0.025
Yes	169 (25.5%)	47 (20.3%)	122 (28.3%)	
No	494 (74.5%)	185 (79.7%)	309 (71.7%)	
**Number of sexual partners in their lives**^d^				0.070
1–2	86 (13.9%)	24 (10.9%)	62 (15.6%)	
3–6	326 (52.8%)	129 (58.6%)	197 (49.5%)	
7 or more	206 (33.3%)	67 (30.5%)	139 (34.9%)	
**Practice of sex for money during their lives**				0.074
Yes	76 (11.2%)	34 (14.3%)	42 (9.6%)	
No	600 (88.8%)	204 (85.7%)	396 (90.4%)	
**Previous children**				0.036
Yes	473 (69.7%)	154 (64.4%)	319 (72.5%)	
No	206 (30.3%)	85 (35.6%)	125 (27.5%)	
**Total**	**681**	**240**	**441**	

^a^ Totals may differ due to the possibility of non-response.

^b^ Value p associated to the test of homogeneity of proportions based on the Pearson chi-squared statistic.

^c^ Mean ± standard deviation = 16.10±2.73 years, 15.56 ± 2.39 years in the group of Women with pregnancies after the diagnosis of HIV∕Aids and 16.40 ±7.61 in the group of Women without pregnancies after the diagnosis of HIV∕Aids.

^d^ The median number of sexual partners throughout life was 5, with total number of partners ranging from 1 to 1300.

The comparison between the groups of women with and without pregnancies after the diagnosis demonstrated that the groups differ in relation to age at diagnosis of HIV/AIDS (p<0.001), level of education (p = 0.002), household income (p = 0.040), use of illegal drugs in their lives (p = 0.001), experience of violence related to the diagnosis of HIV/AIDS (p<0.001), age of first sexual intercourse (p = 0.001), use of condoms in the first sexual intercourse (p = 0.025), and if already had previous children (p = 0.036) (Tables 1 and 2).

The analysis of the group of women with pregnancies after the diagnosis of HIV/AIDS shows that 60.3% of them had the diagnosis of HIV/AIDS at up to 25 years of age (the mean age at the diagnosis was 24,54 ± 6,14 years), 74.2% had a level of education below high school and 64.6% had two or more stable unions in their lives. In this group, 38.1% had already used illegal drugs and 51.3% reported to have suffered violence related to the diagnosis of HIV/AIDS (Table 1). With respect to sexuality and reproductive life, 55.6% had their first sexual intercourse at up to 15 years of age (the mean age at first sex was 15,56 ± 2,39 years), 79.7% had not used condoms in this relationship and 64.4% already had a child before the diagnosis of HIV (Table 2).

Raw Poisson regression models (Table 3) demonstrated a higher occurrence of pregnancies after the diagnosis in women with a lower level of education (RP = 1.44; IC95%: 1.13–1.84); lower income (RP = 1.25 IC95%: 1.01–1.54); with the occurrence of the first sexual intercourse before 15 years of age (RP = 1.40; IC95%: 1.15–1.73); who did not use condoms in their first sexual intercourse (RP = 1.35; IC95%: 1.03–1.76); who used illegal drugs in their lives (RP = 1.43; IC95%: 1.17–1.76); with experience of violence related to the diagnosis of HIV (RP = 1.75; IC95%: 1.44–2.15); and women without previous children (RP = 1.27; IC95%: 1.03–1.56). The lower the age at diagnosis, the higher the occurrence of pregnancies (test of significant linear trend, p <0.001).

**Table 3:** Association between the occurrence of pregnancies after the diagnosis of HIV/Aids and the studied characteristics, Porto Alegre, 2011.

Characteristics	Raw RP (IC95%)*	Adjusted RP (IC95%)*
**Race ∕ethnicity (self-declared)**	**0.188**	**0.500**
White	Reference	Reference
Others	1.15 (0.93–1.40)	1.07 (0.89–1.30)
**Level of education**	**0.003**	**0.029**
< High school	Reference	Reference
≥ High school	1.44 (1.13–1.84)	1.31 (1.03–1.66)
**Household income**	**0.041**	**0.300**
≥ 2 minimum salaries	Reference	Reference
< 2 minimum salaries	1.25 (1.01–1.54)	1.11 (0.91–1.37)
**Number of stable unions**	**0.048**	**0.113**
0	Reference	Reference
1	1.05 (0.63–1.76)	1.04 (0.64–1.71)
≥2	1.37 (0.84–2.24)	1.32 (0.82–2.13)
**Age at first sexual intercourse**	**0.001**	**0.160**
˃15 years	Reference	Reference
≤15 years	1.40 (1.15–1.73)	1.16 (0.94–1.43)
**Use of condom in the first sexual intercourse**	**0.030**	**0.035**
Yes	Reference	Reference
No	1.35 (1.03–1.76)	1.32 (1.02–1.70)
**Use of illegal drugs during their lives**	**<0.001**	**0.166**
No	Reference	Reference
Yes	1.43 (1.17–1.76)	1.16 (0.94–1.42)
**Number of sexual partners in their lives**	**0.063**	**0.043**
1–2	Reference	Reference
3–6	1.16 (0.79–1.69)	0.94 (0.66–1.35)
≥7	1.42 (0.98–2.04)	1.23 (0.88–1.72)
**Age at diagnosis of HIV/Aids**	**<0.001**	**<0.001**
≥ 36	Reference	Reference
31–35	1.79 (0.90–3.24)	1.60 (0.88–2.89)
26–30	3.42 (2.06–5.67)	2.60 (1.53–4.43)
21–25	4.47 (2.75–7.29)	3.18 (1.89–5.35)
≤ 20	5.40 (3.32–8.79)	3.48 (2.02–6.01)
**Violence related to HIV/Aids**	**<0.001**	**0.012**
No	Reference	Reference
Yes	1.75 (1.44–2.15)	1.28 (1.06–1.56)
**Previous children**	**0.026**	**0.193**
Yes	Reference	Reference
No	1.27 (1.03–1.56)	1.15 (0.93–1.43)
**Time with diagnosis of HIV/Aids**	1.09 (1.07–1.11)**	1.05 (1.03–1.07)***

* calculated by Poisson regression;

**value p = 0.001;

***value p<0.001

The adjusted Poisson regression model evidenced that the following variables of exposition maintained an independent effect regarding the occurrence of pregnancies after the diagnosis of HIV: level of education below high school (RP adjusted = 1.31; IC95%: 1.03–1.66), non-use of condoms in the first sexual intercourse (RP = 1.32; IC95%: 1.02–1.70), age at diagnosis of HIV, highlighting women with an age equal to or less than 20 years (RP = 3.48; IC95%: 2.02–6.01); and experience of violence related to the diagnosis of HIV (RP = 1.28; IC95%: 1.06–1.56). In this analysis, the effect of the following variables regarding the outcome was not significant: race/ethnicity, household income, number of stable unions, age in the first sexual intercourse, use of illegal drugs, number of sexual partners in their lives, and the existence of previous children.

In the group of women who got pregnant after diagnosis, the first pregnancy was not planned for 65,4%, and 47,2% reported willingness to undergo a tubal ligation surgery (data not presented in any table).

## Discussion

This study demonstrated that one third of women living with HIV∕AIDS cared for in specialized services in the city of Porto Alegre had at least one pregnancy after the diagnosis. Becoming pregnant after the diagnosis can indicate the exercise of the reproductive right of maternity, especially in a scenario in which the anti-retroviral therapy is universally offered, but can also represent a type of consequence of the situation of vulnerability of women living with HIV/AIDS. The data analyzed here indicates that the pregnancy after diagnosis, for most of the interviewees, indicates the context of vulnerability in which these women find themselves. In this study, post-diagnosis pregnancy is associated with a lower level of education and non-use of condoms in the first sexual intercourse. Furthermore, the lower the age at diagnosis, the more prevalent the outcome. The experience of HIV-related violence was also associated with the occurrence of post-diagnosis pregnancy.

Studies demonstrate that the use of condoms in the population in general is higher between young people particularly regarding their first sexual intercourse [22–25]. Although in the raw analysis the occurrence of pregnancies appears to be associated with the start of the sex life before 15 years old, the adjusted analysis demonstrates that the preponderant factor is the non-use of condoms in the first sexual intercourse. In the studied sample, less than 30% of the women used condoms in their first sexual intercourse, when compared in relation to the occurrence of pregnancies after the diagnosis, the use of condoms fell to 20%. This relates to the gender inequalities which are still significantly present in Latin American countries [10,13,21] and which, in addition to the low education and income levels found in our study, suggest that the women find it hard to negotiate the use of condoms. Despite this finding, there are studies that report that the use of condoms tends to increase after the HIV diagnosis, but condoms are still infrequently or irregularly used [21,26–28].

The age at diagnosis of infection by HIV appears to influence the occurrence of pregnancies. Our results indicate that the lower the age at diagnosis, the higher the occurrence of pregnancies, as already observed in another study [15]. Studies indicate that age is a factor that interferes in the will to conceive [11,27]. Despite several studies reporting that many women living with HIV/AIDS wish to become mothers [8,11,17,19,29–32] our data suggests that the pregnancy after the diagnosis is a result of factors related to the context of vulnerability in which women with HIV/AIDS live.

Although in the multi-varied model the association was not maintained between previous children and the occurrence of pregnancy after the diagnosis of HIV, it is possible that the existence of previous children represents vulnerability in the reproductive trajectory and not the fulfillment of a reproductive right. This hypothesis is sustained by the study of Pilleco [33] which indicates a higher prevalence of induced abortion between women living with HIV/AIDS.

Added to the already described situations of vulnerability is the association found between the occurrence of pregnancies after the diagnosis and violence related to the diagnosis. Several studies have demonstrated that women living with HIV report a higher prevalence of violence during their lives [34,35].

Therefore, the occurrence of pregnancies after the diagnosis of infection by HIV does not indicate, necessarily, the exercise of the reproductive rights by women living with HIV/AIDS, because, as demonstrated, these pregnancies occurred in contexts of vulnerability (low education and income level, non-use of condoms at the first sexual intercourse, significant percentage of women willing to undergo a tubal ligation surgery, and high percentage of unintended pregnancies).

The pregnancy can, in this context, express the difficulties of these women in negotiating with partners about the use of contraceptive methods and the prevention of STD/AIDS and the difficulty in the decisions regarding sexual and reproductive health.

Pregnancy can also be a strategy of social insertion—since maternity performs an important role in the construction of the feminine identity, particularly in Latin American and African societies, and is a way of dealing with the still significantly disseminated prejudice caused by AIDS [17–19].

Taking into account the presented scenario, the interventions of prevention in women’s health should occur earlier, before the start of their sex lives, in order to guarantee the appropriate information and inputs for the prevention of sexually transmitted diseases such as AIDS and unplanned pregnancies. It is fundamental that the health services recognize the contexts of vulnerability of women living with HIV/AIDS, as demonstrated in this study, in order to promote educational actions of reproductive planning that favor the autonomy of women regarding their sexual and reproductive decisions.

## Supporting information

S1 Dataset Women living with AIDS in Porto Alegre. (XLS)

## References

[1] UNAIDS. Global HIV/AIDS Response. Epidemic update and health sector progress towards Universal Access. Report 2011. WHO/UNAIDS/UNICEF. http://www.unAIDS.org/en/media/unAIDS/contentassets/documents/unAIDSpublication/2011/2011130_UA_Report_en.pdf (05 jan 2012, date last accessed).

[2] BRASIL. Boletim Epidemiológico—AIDS e DST Ano III—n° 1—27ª à 52ª semanas epidemiológicas—julho a dezembro de 2013 Ano III—n° 1—01ª à 26ª semanas epidemiológicas—janeiro a junho de 2014).

[3] Connor EM, Sperling RS, Gelber R, Kiselev P, Scott G, O’Sullivan MJ, et al Reduction of maternal infant transmission of human immunodeficiency virus type 1 with zidovudine treatment. New Eng J Med 1994; 331: 1173–1180. 10.1056/NEJM199411033311801 7935654

[4] Newell M-L, Gray G, & Bryson YJ. Prevention of mother-to-child transmission of HIV-1 transmission. AIDS 1997; Suppl 11: 1165–1172. 9451981

[5] Wade NA, Birkhead GS & Warren BL. Abbreviated regimens of zidovudine prophylaxis and perinatal transmission of the human immunodeficiency virus. New Eng J Med 1998; 339(20): 1409–1414. 10.1056/NEJM199811123392001 9811915

[6] UNAIDS. Global plan towards the elimination of new hiv infections among children by 2015 and keeping their mothers alive. 2011. http://www.unAIDS.org/en/media/unAIDS/contentassets/documents/unAIDSpublication/2011/20110609_JC2137_Global-Plan-Elimination-HIV-Children_en.pdf (05 jan 2012, date last accessed).

[7] Feldman R, Maposhere C. Safer sex and reproductive choice: findings from “positive women: voices and choices” in Zimbabwe. Reprod Health Matters 2003; 11(22): 162–173. 14708407 10.1016/s0968-8080(03)02284-5

[8] Paiva V, Santos N, França-Junior I, Filipe E, Ayres JR, Segurado A. Desire to have children: gender and reproductive rights of men and women living with HIV: a challenge to health care in Brazil. AIDS Patient Care and STDs 2007; 21(4): 268–277. 10.1089/apc.2006.0129 17461722

[9] Fiore S, Heard I, Thorne C, Savasi V, Coll O, Malyuta R, et al Reproductive experience of HIV-infected women living in Europe. Hum Reprod 2008; 23(9): 2140–2144. 10.1093/humrep/den232 18567897

[10] Gogna ML, Pecheny MM, Ibarlucía I, Manzelli H, López SB. The reproductive needs and rights of people living with HIV in Argentina: health service users’ and providers’ perspectives. Soc Sci Med 2009; 69(6): 813–820. 10.1016/j.socscimed.2009.06.002 19577833

[11] Marcellin F, Protopopescu C, Abé C, Boyer S, Blanche J, Ongolo-Zogo P, et al Desire for a child among HIV-infected women receiving antiretroviral therapy in Cameroon: results from the national survey EVAL (ANRS 12–116). AIDS Care 2010; 22(4): 441–451. 10.1080/09540120903202913 20140791

[12] Bedimo AL, Bessinger R & Kissinger P. Reproductive choices among HIV positive women. Soc Sci Med 1998; 46(2): 171–179. 9447641 10.1016/s0277-9536(97)00157-3

[13] Barbosa RM & Knauth DR. Esterilização feminina, AIDS e cultura médica: os casos de São Paulo e Porto Alegre, Brasil. Cadernos de Saúde Pública 2003; 19(2): 365–376. 10.1590/s0102-311x2003000800018 15029356

[14] Barnes DB, Murphy S. Reproductive decisions for women with HIV: motherhood’s role in envisioning a future. Qualitative Health Research 2009; 19(4): 481–491. 10.1177/1049732309332835 19299754

[15] Craft SM, Delaney RO, Bautista DT, Serovich JM. Pregnancy Decisions Among Women with HIV. AIDS Behav 2007; 11(6): 927–935. 10.1007/s10461-007-9219-6 17323122 PMC2151976

[16] Knauth DR. Subjetividade Feminina e soropositividade In: Barbosa RM & Parker R (Orgs.). Sexualidades pelo avesso. 1999 São Paulo, Editora 34, pp. 121–136.

[17] Cooper D, Harries J, Myer L, Orner P, Bracken H. “Life is still going on”: Reproductive intentions among HIV-positive women and men in South Africa. Soc Sci Med 2007; 65(2): 274–283. 10.1016/j.socscimed.2007.03.019 17451852

[18] Heard I, Sitta R, Lert F, and The VESPA Study Group. Reproductive choice in men and women living with HIV: evidence from a large representative sample of outpatient attending French hospitals (ANRS-EN12-VESPA Study). AIDS 2007; 21 Suppl 1: S77–S82. 17159592 10.1097/01.aids.0000255089.44297.6f

[19] Aska ML, Chompikul J, Keiwkarnka B. Determinants of Fertility Desires among HIV Positive Women Living in the Western Highlands Province of Papua New Guinea. World Journal of AIDS 2011; 1: 198–207.

[20] Lusti-Narasimhan M, Cottingham J, Berer M. Ensuring the Sexual and Reproductive Health of People Living with HIV: Policies, Programmes and Health Services. Reproductive Health Matters 2007; 15 Suppl 29: 1–3.

[21] Teixeira LB, Pilecco FB, Vigo A, Knauth DR. Sexual and reproductive health of women living with HIV in Southern Brazil. Cad. Saúde Pública, Rio de Janeiro 2013; 29(3):609–620. 10.1590/s0102-311x2013000300018 23532295

[22] Murphy J, Boggess S. Increased Condom Use Among Teenage Males, 1988–1995: The Role of Attitudes, Family Planning Perspectives 1998; 30 (6): 276–280. 9859018

[23] Martinez GM, Abma JC. Sexual Activity, Contraceptive Use, and Childbearing of Teenagers Aged 15–19 in the United States, NCHS Data Brief, No. 209, July 2015 U.S. DEPARTMENT OF HEALTH AND HUMAN SERVICES Centers for Disease Control and Prevention National Center for Health Statistics

[24] Teixeira AMFB, Knauth DR, Fachel JMG, Leal AF. Adolescentes e uso de preservativos: as escolhas dos jovens de três capitais brasileiras na iniciação e na última relação sexual. Cad. Saúde Pública, Rio de Janeiro 2006; 22(7):1385–1396. 10.1590/s0102-311x2006000700004 16791339

[25] Berquó E, Barbosa RM, Lima LP. Uso do preservativo: tendências entre 1998 e 2005 na população brasileira, Rev Saúde Pública 2008;42 Supl 1:34–44. 10.1590/s0034-89102008000800006 18660923

[26] Kline A, Strickler J, Kempf J. Factors associated with pregnancy and pregnancy resolution in HIV seropositive women. Soc Sci Med 1995; 40(11): 1539–1547. 7667658 10.1016/0277-9536(94)00280-7

[27] Peltzer K, Chao LW, Dana P. Family Planning Among HIV Positive and Negative Prevention of Mother to Child Transmission (PMTCT) Clients in a Resource Poor Setting in South Africa. AIDS Behav 2009; 13: 973–979. 10.1007/s10461-008-9365-5 18286365

[28] Njabanou NM, Atashili J, Mbanya D, Mbu ER, Ikomey GM, Kefie CA, et al Sexual Behavior of HIV-Positive Women in Cameroon. Journal of the International Association of Providers in AIDS Care 2011; 12(2) 98–102. 10.1177/1545109711421640 21951727

[29] Sowell RL, Murdaugh CL, Addy CL, Moneyham L, Tavokoli A. Factors influencing intent to get pregnant in HIV-infected women living in the southern USA. AIDS Care 2002; 14(2): 181–191. 10.1080/09540120220104695 11940277

[30] Oladapo OT, Daniel OJ, Odusoga OL, Ayoola-Sotubo O. Fertility desires and intentions of HIV-positive patientes at a suburban specialist center. Journal of the National Medical Association 2005; 97(12): 1672–1681. 16396059 PMC2640756

[31] Steiner RJ, Finocchario-Kessler SS, Dariotis JK. Engaging HIV Care Providers in Conversations With Their Reproductive-Age Patients About Fertility Desires and Intentions: A Historical Review of the HIV Epidemic in the United States. American Journal of Public Health 2013, 103(8):1357–66. 10.2105/AJPH.2013.301265 23763424 PMC4007857

[32] Santos NJS, Barbosa RM, Pinho AA, Villela WV, Filipe EM. Contextos de vulnerabilidade para o HIV entre mulheres brasileiras. Cad. Saúde Públic. 2009; 25 (Supl 2): S321–333. 10.1590/s0102-311x2009001400014 19684939

[33] Pilecco FB, Teixeira LB, Vigo A, Dewey ME, Knauth DR. Lifetime Induced Abortion: A Comparison between Women Living and Not Living with HIV. Plos One 2014; 9(4): e95570 10.1371/journal.pone.0095570 24752119 PMC3994069

[34] Li Y, Marshall CM, Rees HC, Nunez A, Ezeanolue EE, Ehiri JE. Intimate partner violence and HIV infection among women: a systematic review and meta-analysis. J Int AIDS Soc 2014; 17(1): 18845. 24560342 10.7448/IAS.17.1.18845 PMC3925800

[35] Phillips DY, Walsh B, Bullion JW, Reid PV, Bacon K, Okoro N. The Intersection of Intimate Partner Violence and HIV in U.S. Women: A Review. JANAC 2014; 25 (Supl 1): S36–S49. 24216338 10.1016/j.jana.2012.12.006

